# Global assessment of pain care quality for musculoskeletal disorders: Insights from the quality of care index (QCI) from 1990 to 2021

**DOI:** 10.1371/journal.pone.0335235

**Published:** 2025-10-31

**Authors:** Zhicong Xia, Jiangguang Wang, Xia Wen

**Affiliations:** 1 Shenzhen Clinical College of Integrated Chinese and Western Medicine, Guangzhou University of Chinese Medicine, Shenzhen, China; 2 Shenzhen Guangming District People’s Hospital, Shenzhen, China; University of Yaounde I Faculty of Medicine and Biomedical Sciences: Universite de Yaounde I Faculte de Medecine et des Sciences Biomedicales, CAMEROON

## Abstract

**Background:**

Musculoskeletal disorders (MSDs) represent a global primary cause of disability, affecting over 1.69 billion individuals and accounting for over 160 million disability-adjusted life years (DALYs) in 2021. Conventional metrics assess care quality inadequately, necessitating a multidimensional evaluation framework. Thus, this study introduces a novel metric — the Quality of Care Index (QCI) — to comprehensively evaluate the MSD landscape.

**Objective:**

To develop a novel QCI for MSDs and evaluate global trends, disparities, and drivers from 1990 to 2021.

**Methods:**

Using Global Burden of Disease (GBD) 2021 data across 204 countries, we constructed a composite QCI integrating four domains: Pain Relief Rate(PRR), Opioid Use Rate(OUR), Need-Independent Coverage(NIC), and Patient Satisfaction(PS). These were standardized into a single index using Principal Component Analysis (PCA). Trends were analyzed via Estimated Annual Percentage Change (EAPC), with disparities assessed by Socio-Demographic Index (SDI) and geography.

**Results:**

Significant increases in MSDs prevalence (over 12.7% of low back pain; near doubling of osteoarthritis) and DALYs occurred globally, driven by aging populations. Geographic Paradox: Lower- and middle-income countries (LMICs; e.g., Vanuatu, Nicaragua, Central African Republic) achieved higher QCI scores than high-income nations (e.g., Italy, Germany, Japan). QCI improved in LMICs (notably for osteoarthritis/neck pain) but stagnated or even declined in high-income regions. DALYs correlated positively with SDI (R = 0.649–0.863; p < 0.001), with steepest rises in middle- and high-SDI areas for low back pain and osteoarthritis.

**Conclusion:**

The QCI scores reveal critical relationship between MSDs burden and care quality. High-income regions experience challenges of opioid overuse and stagnant patient satisfaction, while LMICs express gains in access and perceived relief. Implementing context-stratified strategies — de-implementing low-value care in high-SDI areas while scaling evidence-based access in resource-constrained regions — constitutes a critical pathway to equitably alleviate the global MSDs burden.

## 1. Introduction

Musculoskeletal disorders (MSDs), including lower back pain (LBP), neck pain(NP), osteoarthritis (OA), rheumatoid arthritis (RA), gout and other musculoskeletal disorders, represent a leading global cause of disability. According to the Global Burden of Disease Study (GBD) 2021, these diseases affected over 1.69 billion individuals [1]. While historically prevalent in high-income regions, MSDs show rising incidence in low- and middle-income countries, particularly in Central Asia [1]. Among BRICS nations, MSDs burden shows steep growth trajectories. In 2021, Brazil and China reported the highest and lowest age-standardized disability-adjusted life years (DALYs), respectively, with women consistently experiencing higher DALYs than men [2]. Within this population, low back pain constitutes the leading contributor to DALYs [3], whereas gout contributes the least [4].

Musculoskeletal disorders frequently accompany other chronic diseases, leading to clinically significant clusters of multimorbidity [5]. These patterns intensify physical disability and decrease quality of life. Public health programs should leverage evidence-based strategies to promote healthy behaviors and mitigate MSD impacts. MSDs’ chronic nature incurs considerable economic costs, both from direct healthcare expenditures and indirect productivity losses. For example, LBP and NP lead to considerable spending on medical and pharmaceutical needs, while increasing simultaneously workplace absenteeism and reducing functional capacity [6]. Employees with musculoskeletal pain exhibit significantly impaired health-related quality of life and work productivity [7]. Addressing the MSD burden requires coordinated policy interventions targeting healthcare delivery optimization and resource allocation [8]. Enhancing the quality of care helps to alleviates patient suffering and also reduces societal costs. Consequently, advancing the measurement and improvement of MSD care quality is key to reducing the global burden.

Conventional metrics — including DALYs, prevalence, and incidence — primarily reflect epidemiological burden rather than care quality. To bridge this gap, we introduce a novel multidimensional QCI for MSDs. This index integrates the following four domains: pain relief rate, opioid use rate, need-independent coverage, and patient satisfaction. By encompassing these dimensions, the QCI delivers a detailed structure for cross-setting quality assessment and informs evidence-based service delivery optimization.

## 2. Methods

### 2.1. Data sources

We utilized estimates from the Global Burden of Disease (GBD) database to obtain global and regional incidence, prevalence, and years lived with disability (YLD) for MSDs. The GBD study is an extensive and internationally recognised work, which annually gathers large amounts of information to estimate the disease burden globally. For instance, the GBD 2021 included a thorough analysis of incidence, prevalence, and YLDs across 371 diseases and injuries in 204 countries [9]. These peer-reviewed GBD studies conform to transparent reporting standards and have been widely used to inform health policy, confirming that using GBD data to assess global incidence, prevalence, and YLD is appropriate and scientific for our analyses.

### 2.2. QCI construction based on four domains

To assess the quality of care for MSDs, we introduce a novel multidimensional QCI integrating four key constructs derived from literature on pain care quality:

**Pain Relief Rate:** Proportion of patients reporting effective relief from chronic musculoskeletal pain after standard treatment. Pain relief is an important aim for musculoskeletal care, and clinical guidelines consider “relieve or reduce suffering and improve patient functioning” to be the goals of effective pain [10]. Prior quality indicator work (e.g., ACP guidelines) underlines that measuring successful pain relief is a valid quality-outcome metric [11].

**Opioid Use Rate:** Proportion of patients receiving opioid therapy for MSDs reflects access and risk of overtreatment. We measured OUR as an indicator of access to potent pain relief. Global studies have measured opioid consumption on a per capita basis and linked low opioid availability to inadequate pain relief in some regions. The Lancet Public Health reports widening disparities in opioid analgesic use, highlighting that countries with low opioid use frequently experience inadequate access to essential pain relief [12].

**Need-Independent Coverage:** Proportion of population receiving pain care services irrespective of disease severity.We incorporated a coverage measure to capture whether necessary care is delivered equitably. This concept aligns with the notion of effective coverage in health systems, which integrates the population’s need for an intervention, the use of that intervention, and its quality. In our context, NIC represents the extent to which individuals receive required musculoskeletal interventions regardless of need barriers. Effective coverage has been recommended for monitoring universal health coverage, explicitly combining need, utilization, and quality into one metric. Our definition of NIC is conceptually consistent with this approach, ensuring that coverage of musculoskeletal treatments is measured not merely by access but by the proportion of need that is met with high-quality care [13].

**Patient Satisfaction:** Proportion of patients reporting satisfaction with pain care delivery and responsiveness. Patient satisfaction is a well-established outcome measure and an independent dimension of care quality. Studies recognize patient satisfaction as reflecting the patient’s perspective on the care experience, and Donabedian included satisfaction as a key healthcare outcome [14].

Data for pain relief rate, opioid use rate, need-independent coverage, and patient satisfaction were synthesized using modeled estimates from GBD proxies (e.g., healthcare access, coverage indices, and surveys), regional literature, and indirect estimation (when direct measures unavailable).

### 2.3. Composite Index via Principal Component Analysis

We standardized the four indicators and applied principal component analysis to identify the main components. The first principal component (PC1), accounting for the most variance across countries, was defined as the QCI. Higher QCI scores reflect a more equitable, balanced, and effective approach to MSDs care delivery. Countries were ranked based on QCI scores for each MSD and throughout the years.

### 2.4. Statistical analyses

The Estimated Annual Percent Change (EAPCP in QCI was calculated from from 1990 to 2021 using log-linear regression.We summarized burden estimates as both crude counts and age-standardized rates (per 100,000) to account for differing population age structures. We calculated EAPC to quantify temporal trends in age-standardized rates. EAPC is defined as the annual rate of change in the log-transformed age-standardized rate and is a standard metric for trend analysis [15]. In practice, we fit a linear regression of the log(rate) over time (y = α + βx + ε), where EAPC = 100×(e^β − 1) [16]. The 95% uncertainty interval (UI) for EAPC was derived from the model’s estimates, enabling inference about increasing or decreasing trends [17]. We also used analysis of variance to test for differences in mean QCI values across categorical subgroups. The analysis of variance assesses whether group means differ more than would be expected by chance, assuming normally distributed residuals and homogeneity of variances.

All analyses were conducted in R (version 4.3.2). We used the FactoMineR package for multivariate data exploration and dimensionality reduction, and ggplot2 for graphics. Statistical Platform for Life Sciences (v1.0) was also used for data cleaning and supplementary plots. Statistical significance was evaluated at the 0.05 level; 95% uncertainty interval are reported for key estimates (e.g., EAPCs). We carefully assessed data completeness. For any missing values in the QCI components, we applied standard imputation or excluded incomplete cases, as appropriate (assuring that small proportions of missing data did not bias results). Outliers were examined graphically and by standardized criteria; extreme values were verified and retained if plausible, since they often reflect true high-burden regions. We conducted sensitivity analyses by, for example, excluding potential outlier countries or using alternative age-standardization weights, to confirm that our main findings were robust. Results of sensitivity checks were consistent with the primary analysis (i.e., no qualitative changes in conclusions). All statistical analyses were conducted by personnel with specialized training in biostatistics and multivariate analysis.

### 2.5. Ethical considerations

All GBD data are publicly available and de-identified. No new patient-level data were collected; thus, ethics approval was not required.

## 3. Results

### 3.1. Global burden of musculoskeletal disorders

The Table 1, S1 and S2 Tables present data on various MSDs (LBP, NP, RA, OA, gout and other musculoskeletal disorders) comparing metrics from 1990 (95% UI) and 2021 (95% UI). The metrics include rate, DALYs number, prevalence number, and incidence number, with statistical significance indicated. The global burden of all six MSDs significantly increased (all P < 0.05) between 1990 and 2021, as detailed in Table 1. For instance, NP incidence rose from 11.44 to 20.42 per 100,000 population, and prevalence increased from 2148.7 to 2610.8 per 100,000 population. LBP exhibited the largest absolute burden, with its prevalence increasing by 12.7%. OA prevalence nearly doubled. Although RA and gout started with lower baselines, they demonstrated steady growth in burden.DALYs also concurrently increased, particularly in high Socio-Demographic Index (SDI) countries, a trend consistent with population aging.

**Table 1:** Comparative analysis of musculoskeletal disorders subtypes in terms of DALYs, incidence and prevalence between 1990 and 2021.

Measure		DALYS		Prevalence		Incidence	
Metric		Rate	Number	Rate	Number	Rate	Number
Neck pain	1990 (95%UI)	11.44 (7.61 ~ 16.33)	214.53 (142.66 ~ 306.25)	114.6 (88.84 ~ 141.52)	2148.66 (1665.67 ~ 2653.36)	24.9 (19.63 ~ 30.67)	466.91 (368.01 ~ 575.11)
	2021 (95%UI)	20.42 (13.64 ~ 28.86)	258.71 (172.83 ~ 365.67)	206.03 (161.76 ~ 252.86)	2610.83 (2049.8 ~ 3204.31)	43.29 (33.94 ~ 52.88)	548.53 (430.11 ~ 670.15)
	Statistically Significant	YES	YES	YES	YES	YES	YES
Rheumatoid arthritis	1990 (95%UI)	1.55 (1.2 ~ 1.98)	28.98 (22.53 ~ 37.08)	7.96 (7.04 ~ 9.09)	149.22 (132.02 ~ 170.34)	0.49 (0.44 ~ 0.55)	9.15 (8.16 ~ 10.23)
	2021 (95%UI)	3.08 (2.31 ~ 3.97)	38.97 (29.28 ~ 50.36)	17.92 (15.97 ~ 20.3)	227.14 (202.41 ~ 257.29)	1 (0.9 ~ 1.11)	12.68 (11.44 ~ 14.12)
	Statistically Significant	No	YES	YES	YES	No	YES
Low back pain	1990 (95%UI)	43.39 (31.08 ~ 58.36)	813.45 (582.79 ~ 1094.1)	386.73 (341.58 ~ 434.16)	7250.82 (6404.31 ~ 8140.14)	165.06 (145.79 ~ 185.93)	3094.78 (2733.33 ~ 3486.07)
	2021 (95%UI)	70.16 (50.19 ~ 94.1)	889.04 (636.07 ~ 1192.5)	628.84 (551.83 ~ 700.88)	7968.7 (6992.9 ~ 8881.64)	266.87 (235.37 ~ 299.41)	3381.84 (2982.63 ~ 3794.11)
	Statistically Significant	YES	YES	YES	YES	YES	YES
Other musculoskeletal disorders	1990 (95%UI)	20.29 (14.42 ~ 27.73)	380.5 (270.28 ~ 519.85)	221.04 (191.78 ~ 254.86)	4144.21 (3595.66 ~ 4778.28)	not applicable	not applicable
	2021 (95%UI)	45.18 (31.73 ~ 61.37)	572.5 (402.1 ~ 777.68)	504.3 (443.7 ~ 574.77)	6390.51 (5622.56 ~ 7283.52)	not applicable	not applicable
	Statistically Significant	YES	YES	YES	YES	not applicable	not applicable
Gout	1990 (95%UI)	0.7 (0.47 ~ 1)	13.08 (8.81 ~ 18.75)	22.26 (17.79 ~ 27.97)	417.44 (333.6 ~ 524.33)	3.98 (3.18 ~ 4.91)	74.68 (59.6 ~ 92.09)
	2021 (95%UI)	1.75 (1.19 ~ 2.48)	22.15 (15.03 ~ 31.48)	56.47 (45.16 ~ 70.29)	715.65 (572.3 ~ 890.7)	9.4 (7.44 ~ 11.73)	119.14 (94.27 ~ 148.67)
	Statistically Significant	No	YES	YES	YES	YES	YES
Osteoarthritis	1990 (95%UI)	8.92 (4.26 ~ 17.98)	167.22 (79.95 ~ 337.18)	256.08 (227.12 ~ 283.44)	4801.18 (4258.26 ~ 5314.18)	20.9 (18.47 ~ 23.1)	391.86 (346.25 ~ 433.18)
	2021 (95%UI)	21.3 (10.19 ~ 42.94)	269.97 (129.12 ~ 544.08)	606.99 (537.87 ~ 670.52)	7691.83 (6815.99 ~ 8496.89)	46.63 (41.12 ~ 51.64)	590.93 (521.1 ~ 654.44)
	Statistically Significant	YES	YES	YES	YES	YES	YES

Abbreviations: DALYs, disability-adjusted life years; UI, uncertainty interval.

Note: The units for Incidence and Prevalence are measured in cases, while DALYs is measured in person-years. The rates for all indicators are expressed per 100,000 population.

### 3.2. QCI trends in various countries around the world

**Fig 1:**
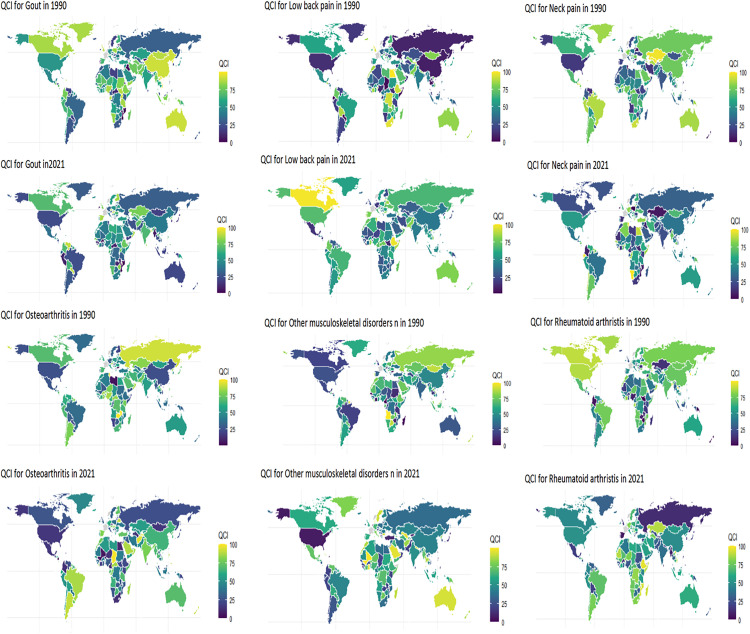
Global distribution of overall QCI for MSDs between 1990 and 2021. Map created by the authors using R (version 4.4) with the ggplot2 (v3.4), sf (v1.0-14) and rnaturalearth (v1.0.0) packages. The base map data is from Natural Earth, where cultural and physical vector data are available under a CC BY 4.0 license and raster data are in the public domain.

Based on the comprehensive visualization of twelve world maps (Fig 1) depicting the Quantity and Complexity Index for six MSDs in 1990 and 2021 — specifically LBP, NP, OA, RA, gout, and other musculoskeletal disorders—this study reveals significant temporal and spatial shifts in global disease burden over the 31-year period. The color-gradient analysis (ranging from light green to dark purple, indicating QCI value) demonstrates a substantial increase in QCI values for many disorders, particularly in low- and middle-income regions (LMICs), alongside notable declines in high-income countries. QCI scores varied markedly by region.

Table 2 presents the top 10 and bottom 10 regions globally for the QCI across six major MSDs. Notably, Italy (ITA) ranked in the bottom 10 for all six conditions, placing last for Gout, LBP, and NP. Conversely, the Central African Republic (CAF), Montenegro (MNE), and Vanuatu (VUT) consistently ranked among the top performers across multiple conditions, with CAF leading in Gout, NP, and RA, and MNE leading in LBP and OA. South Sudan (SSD) also ranked poorly across all six disorders. The analysis revealed geographic disparities, with top-ranking regions concentrated in parts of Africa (CAF, SOM, AGO), the Balkans (MNE), Oceania (VUT), and Central/South Asia (NIC, PAK, IRQ), while the bottom rankings included countries from Europe (ITA, BGR, POL), Africa (SSD, BDI), South America (PRY, URY), North Africa (MAR), and Oceania (TKL). Finland (FIN) exhibited variable performance, ranking well in ‘Other musculoskeletal disorders’ but poorly in RA. Many low-income or fragile states scored high, while high-income countries like Italy and Germany had lower QCI values. Most LMICs showed an increasing QCI for MSDs, particularly for OA and NP, while the inverse pattern held for most of these disorders. In contrast, high-income countries experienced stagnant or declining QCI. The average global QCI increased for RA and gout but remained stable for LBP.

**Table 2:** The top 10 and bottom 10 regions in the global overall QCI ranking.

Gout		Low back pain		Neck pain		Osteoarthritis		Other musculoskeletal disorders		Rheumatoid arthritis	
Location	QCI	Location	QCI	Location	QCI	Location	QCI	Location	QCI	Location	QCI
TOP 10 regions											
CAF	2.937547066	MNE	2.935704798	CAF	2.937069296	MNE	2.939743082	MNE	2.93687504	CAF	2.940530407
VUT	2.931442119	VUT	2.934854294	VUT	2.932817207	VUT	2.931582675	VUT	2.936729744	MNE	2.936839823
MNE	2.922624307	CAF	2.933772687	MNE	2.928150199	CAF	2.928054615	CAF	2.933772687	VUT	2.913382166
NIC	2.888781257	NIC	2.880344787	PAK	2.883340518	NIC	2.88434677	NIC	2.876364694	NIC	2.876877666
PAK	2.875902842	IRQ	2.868034822	NIC	2.881724312	PAK	2.878441914	FIN	2.87542286	PAK	2.874252252
SOM	2.874443064	PAK	2.86713788	IRQ	2.863280328	SOM	2.873960593	IRQ	2.867837861	IRQ	2.86977126
FIN	2.868464897	SOM	2.863560611	FIN	2.860921147	IRQ	2.86667468	SOM	2.866023613	SOM	2.860991711
IRQ	2.857717873	FIN	2.855528888	SOM	2.859920557	FIN	2.86330478	PAK	2.862285474	PER	2.851139181
PER	2.851378083	PER	2.853097593	PER	2.850273911	PER	2.851654991	AGO	2.856677557	FIN	2.849989427
GNQ	2.846826141	GNQ	2.846572191	AGO	2.842201084	AGO	2.848147024	PER	2.855863995	GNQ	2.843020872
Bottom 10 regions											
ITA	−2.954190578	ITA	−2.956402053	ITA	−2.963766409	ITA	−2.946505799	ITA	−2.944125025	ITA	−2.945784058
SSD	−2.918860528	SSD	−2.935006243	SSD	−2.930698354	SSD	−2.94450915	SSD	−2.930895651	SSD	−2.939764624
TKL	−2.906034358	TKL	−2.907539746	TKL	−2.900258847	TKL	−2.906575183	BGR	−2.905932235	TKL	−2.909992026
CUB	−2.899494421	BGR	−2.902459806	BGR	−2.897531198	BGR	−2.902756233	TKL	−2.898916165	BGR	−2.899254488
BGR	−2.89571921	CUB	−2.888823119	CUB	−2.884737908	CUB	−2.887901109	CUB	−2.89005909	CUB	−2.893929631
TKL	−2.868884793	TKL	−2.880291977	BDI	−2.863798531	TKL	−2.862873094	BDI	−2.859026029	URY	−2.866516218
URY	−2.857930543	PRY	−2.863053186	URY	−2.856136676	URY	−2.86284816	URY	−2.85614261	TKL	−2.856132565
PRY	−2.850977593	POL	−2.858710002	TKL	−2.854192023	POL	−2.860509309	PRY	−2.855947378	MAR	−2.853672791
POL	−2.847422203	URY	−2.854839923	POL	−2.852483664	BDI	−2.857246779	POL	−2.851431413	BDI	−2.851962615
MAR	−2.84245245	BDI	−2.8518147	MAR	−2.842114898	PRY	−2.849892459	MAR	−2.849665575	PRY	−2.847722191

**Abbreviations:** CAF, the Central African Republic; MNE, Montenegro; VUT,Vanuatu; NIC, Nicaragua; PAK, Pakistan; IRQ, Iraq; SOM, Somalia; FIN, Finland; PER, Peru; GNQ, Equatorial Guinea (Guinée Équatoriale); AGO, Angola; ITA, Italy; SSD, South Sudan; TKL, Tokelau; CUB, Cuba; BGR, Bulgaria; PRY, Paraguay; POL, Poland; URY, Uruguay; MAR, Morocco; BDI, Burundi.

### 3.3. The association between the burden of MSDs and SDI

**Fig 2:**
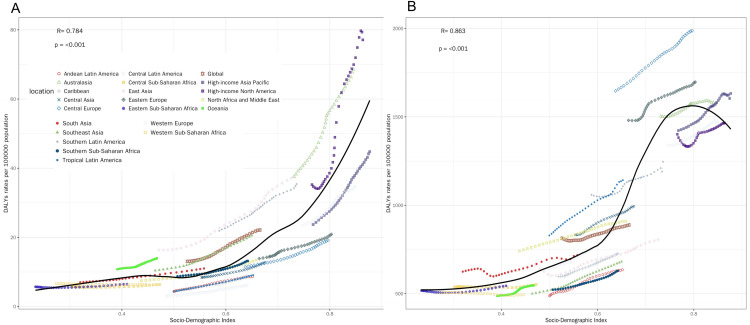
Correlation between DALYs and SDI. A is correlation between DALYs and SDI of gout; B is correlation between DALYs and SDI of LBP.

**Fig 3:**
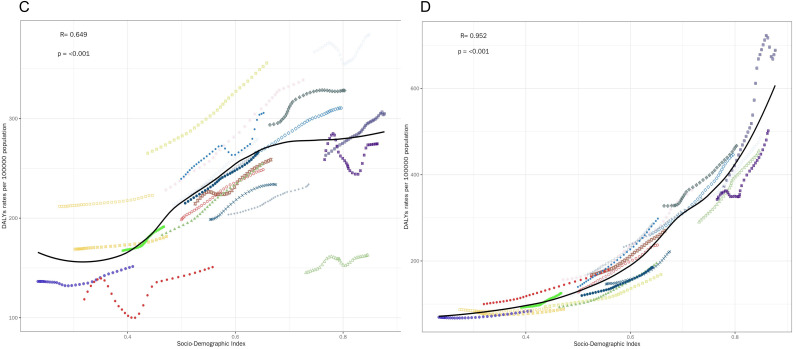
Correlation between DALYs and SDI. C is correlation between DALYs and SDI of NP; D is correlation between DALYs and SDI of OA.

**Fig 4:**
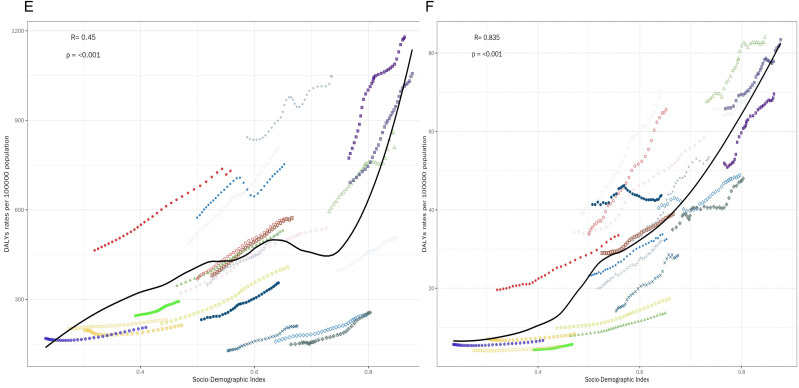
Correlation between DALYs and SDI. E is correlation between DALYs and SDI of other musculoskeletal disorders; F is correlation between DALYs and SDI of RA.

Our findings show the correlation of DALYs rate (per 100,000 population) with SDI for different MSDs types divided into 6 subplots (Figs 2–4). The key observations are detailed as follows: The DALYs rate of gout (Fig 2A) showed a significantly positive correlation with SDI (R = 0.784, p < 0.001). The LOESS curve revealed that as SDI increased, the DALYs rate of gout exhibited an accelerating upward trend, with a particularly drastic surge in the high – SDI range (SDI > 0.8). The DALYs rate of LBP (Fig 2B) had an even stronger positive correlation with SDI (R = 0.863, p < 0.001). The LOESS curve demonstrated rapid growth in the middle- to high- SDI interval (SDI ≈ 0.6–0.9). The correlation between the DALYs rate of NP (Fig 3C) was relatively weaker (R = 0.649, p < 0.001). The LOESS curve displayed a first flat then rising pattern. The DALYs rate of OA (Fig 3D) showed an extremely strong positive correlation with SDI (R = 0.952, p < 0.001), and the LOESS curve was nearly a monotonically steep ascent. Although there was still a significantly positive correlation (R = 0.45, p < 0.001), the correlation between SDI and the DALYs rate of other musculoskeletal disorders (Fig 4E) was the lowest among all the conditions analyzed. The DALYs rate of RA (Fig 4F) was significantly positively correlated with SDI (R = 0.835, p < 0.001). The LOESS curve showed rapid growth in the middle- to high- SDI interval (SDI ≈ 0.5–0.9).

**Fig 5:**
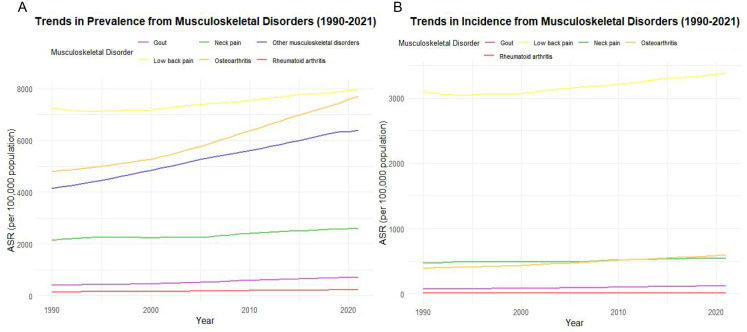
Trends in the prevalence and incidence of MSDs from 1990 to 2021.

Fig 5A shows trends in age-standardized prevalence (ASR per 100,000 population) over time for different types of MSDs. From 1990 to 2021, the prevalence of all MSDs showed an upward trend, with LBP (yellow) and OA (orange) increasing most significantly, while RA (red) and gout (purple) increasing more slowly. Fig 5B shows the change in age-standardized incidence rate (ASR per 100,000 population) over time for the same MSDs type. Incidence is slowly increasing overall, with LBP (yellow) remaining the most significant driver, while RA (red) and gout (purple) remain low. In summary, Fig 5 suggest that LBP and OA are the most important public health problems in MSDs, suggesting that medical resources should prioritize these diseases.

**Fig 6:**
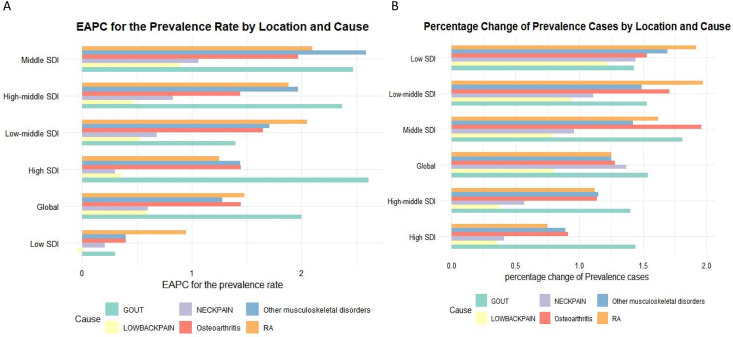
Prevalence and case changes stratified by SDI.

Fig 6A shows the rate of change in prevalence for each MSDs type stratified by SDI (low SDI to high SDI). Low SDI regions have lower EAPC (close to 0 or negative), while high SDI regions show moderate positive growth (around 0.5–1%). LBP and OA varied significantly across all SDI levels. Fig 6B shows the percentage change in the prevalence of MSDs between 1990 and 2021, stratified by SDI. The lowest variation (about 1.5–2%) was in the low SDI and low-medium SDI regions, and the smallest change (about 0.5–1%) in the high SDI region. Low back pain and osteoarthritis contribute the most.

## 4. Discussion

### 4.1. Principal findings and comparison with existing literature

This study introduces a novel metric-QCI- for a more comprehensive evaluation of global MSDs care than is possible with traditional metrics like prevalence or DALYs alone. Our study, covering 204 countries from 1990 to 2021, discovers three primary findings: an ongoing increase in the global burden of MSDs, a significant but paradoxical disparity in care quality between high- and lower-income countries, and divergent drivers of care quality depending on SDI level. First, our findings verify the escalating global burden of MSDs, as the occurrence of issues such as LBP and OA rising substantially over the past three decades. This tendency is in line with previous GBD reports, which have consistently recognized MSDs as the leading cause of disability worldwide [18]. The observation of a strong positive link between DALYs and the SDI underscores the profound effects of population aging and the global shift towards chronic non-communicable diseases [19]. The rise in DALYs in regions with high SDI might reflect enhanced diagnostic capabilities, yet they also signal a growing strain on health systems struggling to manage long-term disability [20]. Our study’s most striking finding is the “geographic paradox,” where several LMICs, such as Vanuatu and Nicaragua, outperforming developed countries like Italy, Germany, and Japan in QCI scores. This counterintuitive finding contradicts the conventional assumption that more health expenditure and advanced technology automatically equate to higher quality care. Our QCI framework, by integrating domains like OUR and PS, helps deconstruct this paradox. The stagnant or declining QCI scores in high-income countries appears to be driven by factors symptomatic of overmedicalization, including high rates of opioid use and faltering patient satisfaction. This is consistent with an increasing amount of literature criticizing the low-value care delivered for chronic MSDs in affluent countries, where a biomedical emphasis on imaging and interventions neglects the complex biopsychosocial nature of chronic pain [21,22]. The opioid crisis, particularly in North America, stands as a stark example of how well-intentioned pain management strategies can lead to iatrogenic harm, a factor our QCI correctly penalizes [23].

Conversely, the improving QCI scores in many LMICs, particularly for OA and NP, suggest progress in fundamental aspects of care. These gains are likely attributable to improvements in access, as reflected in the NIC domain, and potentially higher perceived pain relief. While it may be argued that higher satisfaction in these regions is due to lower patient expectations, it may also be a sign of reflect care models that are more community-integrated and less reliant on pharmacological escalation [24]. Nevertheless, the lower DALYs reported in low-SDI regions may also point to significant under-diagnosis and data scarcity, a persistent hurdle in global health research [25].

Our findings carry significant implications for global health policy. The QCI score demonstrates that a “one-size-fits-all” approach to asserting MSD care quality is not available. In high-SDI countries, the priority must be to moving away from low-value interventionist paradigms and control opioid abuse. Instead, resources ought to be shifted towards evidence-based, multidisciplinary pain management programs that incorporate physical therapy, psychological support, and patient education [26,27]. For LMICs, it is essential to focus on strengthening health systems to expand equitable access, enhance diagnostic capabilities, and ensure that care is delivered in a culturally appropriate and sustainable manner. The QCI serves as a vital tool for ministries of health to benchmark progress and identify specific domains needing improvement.

Our findings are broadly consistent with prior analyses of the global burden of MSDs. The upward trends in disease prevalence and YLDs mirrored those reported by the GBD Collaborators [9,28]. For example, previous systematic analyses highlighted rising prevalence of LBP and OA as leading contributors to musculoskeletal disability [29,30], and our results similarly showed these conditions dominate the burden. The regional disparities we document concur with literature on health inequalities: many studies have found that wealthier countries have greater per-capita opioid consumption and wider healthcare coverage [12,16]. Compared to these prior works, our study integrates multiple dimensions into a single index, offering a more holistic assessment. However, our analysis also highlights new patterns. For instance, the slowing improvement of PRR in some high-income countries contrasts with assumptions that pain management was already optimal. Likewise, the relatively high patient satisfaction scores in certain regions (despite moderate OUR) suggests areas where non-opioid management may be effective, an aspect less emphasized in earlier work.

### 4.2. Strengths and limitations of the study

This study has several strengths. For the first time, a composite quality index for MSDs is used globally, synthesizing four distinct care domains using comprehensive GBD data. This method goes beyond simple burden measurement to provide practical insights into health system performance. However, the limitations of the study need to be acknowledged. Modeled estimates and proxies from the GBD study were used to construct the QCI domains, particularly for patient satisfaction and pain relief, due to the lack of standardized primary data in all countries. The ecological nature of our country-level analysis precludes inferences about individual patient experiences and is subject to the inherent limitations of GBD modeling [31].

### 4.3. Research gaps and future directions

Our study points to several gaps and avenues for future work. First, more granular data on pain management practices (including non-opioid therapies) and patient-reported outcomes for musculoskeletal conditions are needed to refine quality metrics. Future research should validate the QCI against clinical outcomes or more detailed patient data. Second, the reasons for regional disparities deserve deeper investigation: mixed-methods studies could examine how health system factors (e.g., insurance coverage, provider availability) and cultural attitudes impact each QCI dimension. Longitudinal monitoring of QCI could assess the effects of policy changes or interventions (such as opioid regulation or expanded insurance). Third, expanding QCI to subnational analyses would be valuable, as within-country inequities (e.g., rural vs. urban) can be large; this will require better local data. Finally, similar QCI frameworks could be developed for other disease areas, providing a standardized tool to compare care quality internationally.

Overall, while this study advances understanding of global musculoskeletal care, ongoing efforts should aim to obtain higher-quality data, refine the QCI with clinical validation, and explore interventions that can close the identified quality gaps.

## Supporting information

S1 Table GBD dataset (1). (CSV)

S2 Table GBD dataset (2). (CSV)
